# Population genetic structure of tropical bed bug (Hemiptera: Cimicidae) populations and their breeding pattern in Iraq

**DOI:** 10.1093/jisesa/ieae010

**Published:** 2024-02-16

**Authors:** Hussein Ali Baqir, Abdul Hafiz Ab Majid

**Affiliations:** Household & Structural Urban Entomology Laboratory, Vector Control Research Unit, School of Biological Sciences, Universiti Sains Malaysia (USM), 11800 Minden, Malaysia; Department of Plant Protection, Agriculture College, University of Kerbala, 56001 Karbala, Iraq; Household & Structural Urban Entomology Laboratory, Vector Control Research Unit, School of Biological Sciences, Universiti Sains Malaysia (USM), 11800 Minden, Malaysia; Centre for Insect Systematics, Faculty of Science and Technology, Universiti Kebangsaan Malaysia (UKM), 43600 Bangi, Malaysia

**Keywords:** tropical bed bug, microsatellite marker, gene flow, genetic difference

## Abstract

A study was conducted to investigate the population genetic structure and breeding pattern of 140 tropical bed bugs, *Cimex hemipterus* (F.) (Hemiptera: Cimicidae), collected from 14 infested sites in major cities in Iraq. The samples were genotyped using a set of 7 polymorphic microsatellite markers. High genetic variety was seen among populations, with an average of 2–9 alleles per locus. The number of alleles across 7 microsatellite loci was between 6 and 18. There was a notable disparity in the alleles per loci when comparing the overall population to those within it. The overall population exhibited an average observed heterozygosity of 0.175 and an average expected heterozygosity of 0.730. Among the population, the average observed heterozygosity was 0.173, while the average expected heterozygosity was 0.673. Analysis of molecular variance (AMOVA) revealed that 93% of the genetic variability was within the populations, and 7% was among them. The genetic differentiation coefficient (*F*_ST_ = 0.045), indicates a low degree of genetic differentiation and a high degree of inbreeding (*F*_IS_ = 0.761), as indicated by notably significant positive inbreeding coefficients. Admixed individuals were revealed using STRUCTURE and neighbor-joining phylogenetic trees, demonstrating moderate gene flow between populations and a lack of genetic structure in the regional groups. Thus, both active dispersion and human-mediated dispersion possess the potential to influence the low population genetic structure of tropical bed bug *C. hemipterus* populations in Iraq, which can have implications toward tropical bed bug and management strategies.

## Introduction

*Cimex hemipterus*, also known as tropical bed bugs, is an ectoparasite that primarily feeds mostly on human blood during the night. This species has emerged as a noteworthy global public health concern in recent times (Akhoundi et al. 2023). Several factors, such as insecticide resistance, increasing international travelers, trading secondhand items, and ineffective control strategies, may contribute to the global resurgence of bed bugs (Cooper et al. 2015, Raab et al. 2016, Alizadeh et al. 2019).

Bed bug eradication is costly as a result of the difficulties in implementing control strategies, which facilitate the extensive dissemination of bed bugs globally (Akhoundi et al. 2016). At the local scale, the bed bugs associated with humans are highly dependent on the mobility patterns exhibited by human hosts. They may use household items such as furniture, clothing, soiled bedding, bags, and other household items for makeshift conveyances. Bed bugs can also spread in hotel or apartment building rooms by using cracks in walls or gaps around plumbing or ventilation systems (Wang et al. 2010, Booth et al. 2012). Tropical bed bug *C. hemipterus* biology, including infestation dynamics and prevalence, remains unexplored in Iraq. As bed bugs continue to expand globally, the impact of active dispersal compared to passive dispersal (e.g., human-mediated) on the genetic structure of tropical bed bug *C. hemipterus* populations in Iraq is still unknown. Consequently, it is becoming increasingly necessary to gain a comprehensive understanding of the basic biology within these populations, and this knowledge will form the essential basis for designing effective management strategies to eradicate this pest.

The genetic structure of bed bug *C. lectularius* has been investigated in numerous studies (Booth et al. 2012, Saenz et al. 2012, Fountain et al. 2014, Akhoundi et al. 2015, Narain et al. 2015, Raab et al. 2016). However, limited molecular studies have investigated the genetic structure of the tropical bed bug *C. hemipterus* using microsatellite markers (Seri Masran and Ab Majid 2019, Wan Mohammad et al. 2020).

In general, knowledge obtained from microsatellite analysis enables the determination of genetic differences and relatedness within and between populations (Sunnucks 2000, Zhang and Hewitt 2003, Avise 2004, Lee et al. 2008, Gardner et al. 2011, Betson et al. 2014). To date, the genetic structure and breeding patterns of tropical bed bug *C. hemipterus* have not been studied in Iraq. As an initial step in this endeavor, we used species-specific microsatellite markers to explore the genetic structure of the tropical bed bug *C. hemipterus* in Iraq. These markers were utilized to assess gene flow and genetic differentiation among the tropical bed bug populations sampled.

## Materials and Methods

### Sample Collection

**Fig. 1.**
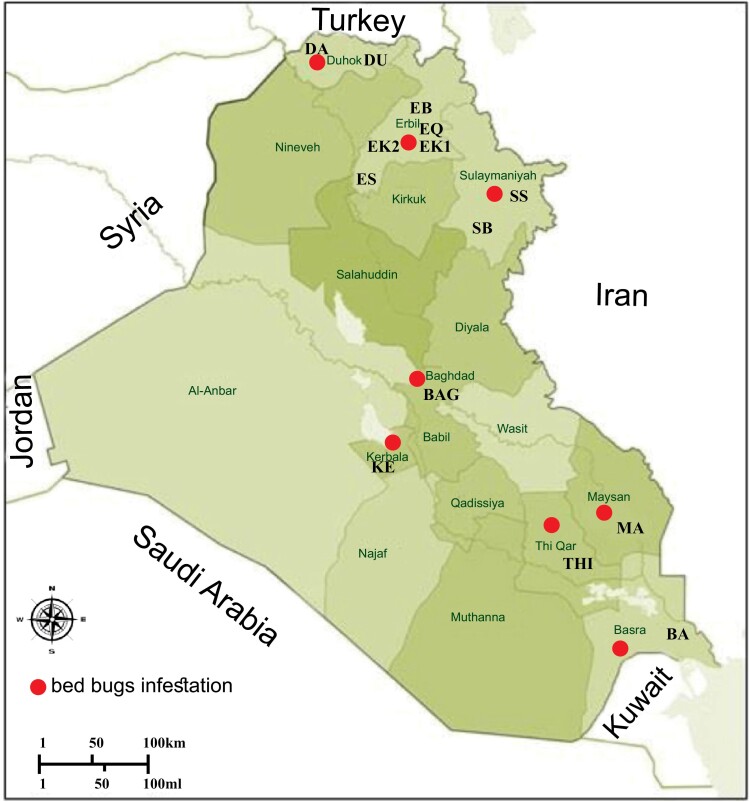
The sampling locations of tropical bed bug *C. hemipterus* in Iraq are shown on the map. Table 1 contains abbreviations for collection.

Tropical bed bugs were collected from various infested sites in urban and suburban locations across Iraq between 2020 and 2021 (Fig. 1, Table 1). Genomic DNA was individually extracted from 10 adult specimens randomly selected at each of the 14 infested sites, using the Real Genomic DNA Extraction Kit Mini (RBC Bioscience, Taiwan). The NanoDrop was used to determine the concentration of DNA of each specimen and the DNA purity range of (1.80–2.00) to proceed with PCR amplification using microsatellite markers.

**Table 1.** Details to collecting tropical bed bug samples from sleeping beds in major Iraqi cities

Governorate	City	Abbreviation	Collection date	Latitude and longitude
Erbil	Kasnazan	EK1	2020	36.12 N 44.154 E
Erbil	Khatun Awa	EK2	2020	36.173 N 44.055E
Erbil	Serbest	ES	2020	36.193 N 43.951E
Erbil	Baharka Road	EB	2021	36.258 N 40.011E
Erbil	City center	EQ	2021	36.189 N 44.007 E
Sulaimani	City center	SS	2021	35.566 N 45.396E
Sulaimani	Bazian sub-district	SB	2021	35.603 N 45.131E
Duhok	City center	DA	2021	37.167 N 42.669E
Duhok	City center	DU	2021	37.167 N 42.668E
Baghdad	Al-Kadhimiya	BAG	2021	33.37 N 44.338E
Kerbala	AL-Hussainiya sub-district	KE	2021	32.629 N 44.057E
Maysan	Amara District	MA	2021	31.845 N 47.131E
Thi Qar	City center	THI	2021	31.047 N 46.262E
Basra	City center	BA	2021	30.458 N 47.813E

The genotyping was performed on 10 individuals from each of the 14 locations using 7 microsatellites’ markers, namely (Ch_01dn, Ch_02dn, Ch_13dn, Ch_16trn, Ch_35trn, Ch_09ttn, Ch_14ttn) (Table 2), as described by Seri Masran and Ab Majid (2019). The PCR was performed using a Thermal Cycler (TaKaRa, Japan). The PCR reaction contains a total volume of 25 μl, compromising 12.5 μl of green buffer (EconoTaq, Lucigen, USA), 0.5 μl of each primer, 8 μl DNA template, and 3.5 μl ddH_2_O. The PCR cycling requirements specified in the program are as follows: initial denaturation carried out at 95 °C for 3 min, followed by 35 cycles of 94 °C for 30 s (denaturation), annealing at 45 °C for 30 s, 72 °C for 1 min (extension), and a final extension step at 72 °C for 5 min.

**Table 2.** Characteristics of 7 microsatellites developed for bed bug *C. hemipterus*

Locus	Repeat	Primer sequences (5ʹ–3ʹ)	Allele size	Accession number
Ch_01dn	(AT)_6_	F = CAACACCGCATCCTAGC R = CCTGGCCTGGCAATCTG	346	Pr032816815
Ch_02dn	(AT)_8_	F = CCGCCACCTTGAATTGGAC R = GTCGGGACCTCCTTGATCC	334	Pr032816816
Ch_13dn	(AT)_8_	F = CGATTGTAAATCCCGAGCC R = CCGCTGATCTACCCATTTG	255	Pr032816827
Ch_16trn	(ATT)_5_	F = CTTGACATGGAAGACACGGC R = ATCATCTTCAGGGCTCAAAG	350	Pr032805544
Ch_35trn	(AAG)_4_	F = GGACGTTAAAGGGAAAGTGC R = GTTTGTCACCAGCCATCGG	436	Pr032805563
Ch_09ttn	(GATT)_4_	F = ACACCGATGCACAGAGTTTC R = CTGCGGAGCAACATTAGCC	255	Pr03285531
Ch-14ttn	(AGAT)_4_	F = ACGAGGCGGTGATTTAAGG R = GGAGTCCGGGAGTGGATCTG	274	Pr032805541

### Analysis of Genetic Data

The GENEPOP software v.4.0 (Raymond and Rousset 1995, Rousset 2008) and Micro-Checker v.2.2.3 (Van Oosterhout et al. 2004) were utilized to check null alleles, scoring errors, and allelic dropouts. The genepop files were converted to structure format using PGDSpider version 2.0.8.3 (Lischer and Excoffier 2012). FSTAT v2.9.3.2 was employed to compute the *F*_ST_ (genetic differentiation)-based *F*_IS_ (inbreeding coefficient) between the sampled populations. Each pair and the coefficient of relatedness (r) were carried out using FSTAT (Goudet 2002). CERVUS 3.0.7 software was applied to calculate polymorphic information criteria (PIC) for every location (Kalinowski et al. 2007). The population genetic distances were determined using POPTREEW software and an unrooted neighbor-joining (NJ) tree with 1,000 bootstrap replicates (Takezaki et al. 2010).

The genetic structure of populations was determined using STRUCTURE version 2.2 (Falush et al. 2003) to group all individuals into clusters displaying their mixing. The program was running with 10 runs for each possible value of K (number of genetic clusters), and with a burn-in period of 100,000 and 100,000 Markov Chain Monte Carlo steps. The value of K was determined to be 15 (Evanno et al. 2005). STRUCTURE HARVESTER was utilized to estimate the most likely number of clusters in the dataset (Earl and VonHoldt 2012). Finally, Cluster Markov Packager across K (CLUMPAK) was used to generate a structure result analysis graphical representation (Kopelman et al. 2015). The GenAlEX v.6.5 program was utilized to assess the genetic differentiation between populations by analysis of molecular variance (AMOVA) (Peakall and Smouse 2012). In addition, Bottleneck v1.2.02 software was used to evaluate the potential for a bottleneck effect (Piry et al. 1999).

## Results

A total of 140 individuals from 14 populations were successfully genotyped using 7 microsatellite loci. The electropherogram obtained from fragment analysis showed peaks in all samples. The 7 microsatellite loci exhibited polymorphism in all populations, with alleles ranging from 6 to 18 per locus (Table 3). The mean observed heterozygosity (*H*_obs_) and expected heterozygosity (*H*_exp_) were 0.730 and 0.175, respectively (Table 4). The average *H*_obs_ was observed to be lower than the average *H*_exp_. PIC values greater than 0.5, the mean PIC value was 0.705 (Table 4).

**Table 3.** Details regarding the number of alleles per each population of bed bug *C. hemipterus* from Iraq for 7 microsatellites loci.

Population	Locus								
	Ch-01dn	Ch-02dn	Ch-13dn	Ch-16trn	Ch-35trn	Ch-09ttn	Ch-14ttn	Mean	Standard deviation
EK1	9	4	3	7	4	3	4	4.86	2.27
EK2	4	7	3	3	5	6	4	4.86	1.51
ES	7	4	2	5	5	3	4	4.93	1.60
EB	5	5	3	3	3	3	5	4.88	1.07
EQ	5	8	4	5	3	5	7	4.96	1.70
SS	6	3	3	4	4	6	6	5.18	1.40
SB	5	6	3	4	4	3	4	5.26	1.07
DA	5	4	4	4	4	3	6	4.99	0.95
DU	5	7	4	3	4	3	2	5.06	1.63
BGA	6	4	3	4	5	3	6	7.07	1.27
KE	5	4	3	4	4	3	5	6.30	0.82
MA	6	5	5	3	3	4	4	5.42	1.11
THI	7	5	4	3	4	2	5	5.90	1.60
BA	6	4	3	4	4	4	4	5.61	0.90
							Mean	4.85	
Mean	5.79	5.00	3.36	4.00	4.00	3.64	4.71	4.85	
Standard deviation	1.25	1.47	0.74	1.11	0.68	1.22	1.27		
Total number	18	17	6	12	8	12	14	12.4	

**Table 4.** Details information regarding comparison across all 7 microsatellites. Number of Alleles (Na), observed heterozygosities (*H*_obs_), expected heterozygosities (*H*_exp_), polymorphism information content (PIC), and Hardy–Weinberg Equilibrium (HWE)

Locus	NA	*H*_exp_	*H*_obs_	PIC	HWE
Ch_01dn	18	0.824	0.241	0.800	*P* < 0.05
Ch_02dn	17	0.729	0.229	0.692	*P* < 0.05
Ch_13dn	6	0.581	0.150	0.527	*P* < 0.05
Ch_16trn	12	0.816	0.014	0.790	*P* < 0.05
Ch_35trn	8	0.775	0.250	0.736	*P* < 0.05
Ch_09ttn	12	0.682	0.079	0.626	*P* < 0.05
Ch-14ttn	14	0.703	0.264	0.761	*P* < 0.05
Mean	12.48	0.730	0.175	0.705	Significant

Based on the analysis among 14 populations, the average observed heterozygosity deviated by 0.173 from the expected value of 0.673 (Table 5).The average fixation index inbreeding coefficient (*F*_IS_) for all populations was 0.755. The mean value of genetic differentiation among all populations was low (*F*_ST_ = 0.045, 95% CI = 0.009–0.079) (Table 5). The inbreeding coefficient *F*_IS_ value was 0.761 (95% CI = 0.681–0.825). The mean relatedness coefficient of *r* was found to be 0.051 (95% CI = 0.011–0.087).

**Table 5.** Information regarding the observed heterozygosity (*H*_obs_), expected heterozygosity (*H*_exp_), and fixation index inbreeding coefficient (*F*_IS_) for each of all the 14 sampled, and summary of *F* statistics values for 95% confidence intervals grouped as the region and overall population

Population	*H*_obs_	*H*_Exp_	*F*_IS_	Region	*F*_ST_(CI)	*F*_rt_(CI)	*F*_IS_(CI)	r(CI)
EK1	0.243	0.644	0.636	North	0.053 (0.020-0.086)	0.782 (0.699-0.870)	0.769 (0.685-0.859)	0.060 (0.024-0.093)
EK2	0.186	0.622	0.713					
ES	0.129	0.669	0.837					
EB	0.186	0.685	0.739					
EQ	0.186	0.741	0.760					
SS	0.143	0.699	0.804					
SB	0.186	0.670	0.734					
DA	0.143	0.703	0.805					
DU	0.071	0.623	0.891					
BAG	0.257	0.682	0.636	Capital and Mid-Euphrates	0.215 (0.132–0.301)	0.810 (0.609–0.952)	0.754 (0.529–0.938)	0.239 (0.155–0.324)
KE	0.157	0.642	0.765					
MA	0.214	0.654	0.684	South	0.017 (0.039–0.074)	0.710 (0.521–0.848)	0.703 (0.524–0.837)	0.022 (0.042–0.062)
THI	0.186	0.717	0.751					
BA	0.129	0.677	0.818					
**Means**	**0.173**	**0.673**	**0.755**	**Iraq**	0.045 (0.009–0.079)	0.772 (0.691–0.863)	0.761 (0681–0.852)	0.051 (0.011–0.087)
**Overall**								

**Fig. 2.**
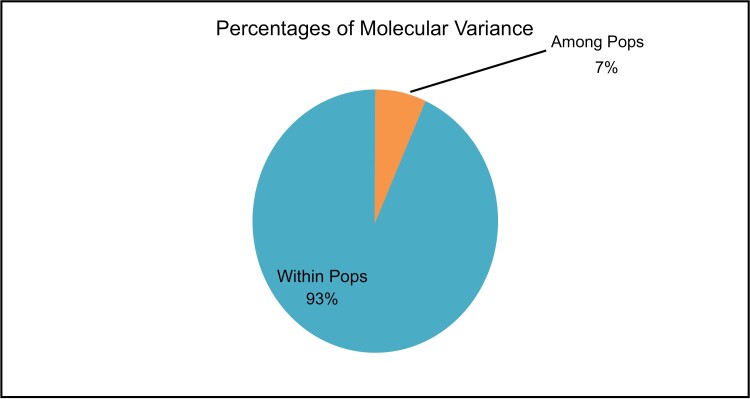
Variation in molecular diversity within and among tropical bed bug *C. hemipterus* populations in Iraq through the comparable percentages.

**Fig. 3.**
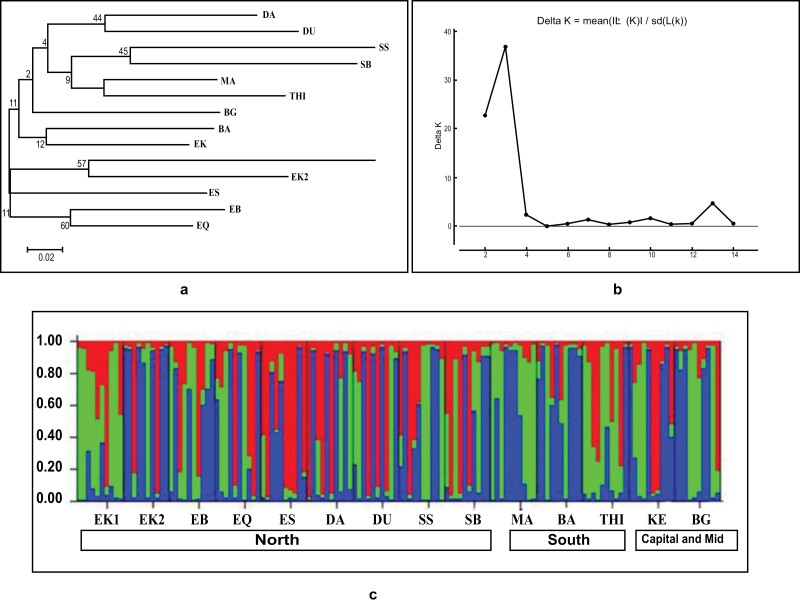
A) Elucidating the genetic distance of all tropical bed bug populations by either neighbor-joining (NJ) tree. Presented below the tree are the bootstrap values and a scale for the distance. The list of abbreviations can be found in (Table 1). B) Bayesian analysis showed 3 clusters with a DeltaK value of *K* = 3. C) The tropical bed bug *C. hemipterus* population structure in Iraq shows 3 clusters. Information is depicted graphically with *K* = 3 clusters as the most probable. A different color represents each cluster and each individual with a vertical bar.

The molecular variance analysis (AMOVA) revealed that 93% of the variation was found within populations. Conversely, only 7% of the molecular variance was found among the populations. This result indicated a low genetic differentiation observed in the population of tropical bed bug *C. hemipterus*, implying low gene flow due to inbreeding (Fig. 2). To estimate the genetic distance, NJ was used. The results obtained from the NJ tree showed that the 14 populations can be grouped into 3 clusters with high bootstrap values (Fig. 3A).

Bottleneck analysis assessed recent bottleneck events, which indicated shift in 12 out of 14 populations (Table 6). The evidence of bottleneck events was assessed by excess homozygosity in the populations. This could be due to a decrease in effective population size of tropical bed bug due to control activities and the speed at which the population recovered (Freeland 2005).

**Table 6.** Bottleneck analysis within 14 population of *C. hemipterus* in Iraq, and the percentage of individuals belonging to each of the 3 inferred clusters is determined by DK (Fig. 3), as determined by the Bayesian clustering methods STRUCTURE

Population	Infinite allele model	Stepwise mutation model	Two phase mutation model	Mode shift	Inferred clusters		
					1	2	3
EK1	0.320	0.172	0.202	Normal	0.267	0.633	0.101
EK2	0.016	0.272	0.088	Shifted	0.199	0.131	0.670
ES	0.151	0.178	0.270	Normal	0.596	0.084	0.320
EB	0.033	0.003	0.055	Shifted	0.247	0.367	0.386
EQ	0.575	0.037	0.499	Shifted	0.330	0.291	0.380
SS	0.151	0.039	0.488	Shifted	0.279	0.313	0.407
SB	0.555	0.044	0.441	Shifted	0.368	0.274	0.358
DA	0.403	0.015	0.284	Shifted	0.396	0.206	0.398
DU	0.410	0.015	0.411	Shifted	0.344	0.249	0.406
BAG	0.424	0.029	0.400	Shifted	0.138	0.391	0.470
KE	0.410	0.051	0.377	Shifted	0.302	0.242	0.456
MA	0.572	0.099	0.313	Shifted	0.044	0.535	0.422
THI	0.024	0.001	0.018	Shifted	0.297	0.522	0.180
BA	0.605	0.024	0.314	Shifted	0.099	0.233	0.668

The STRUCTURE software was used to analyze the population structure of tropical bed bug *C. hemipterus*. The high values of LnP (*K*) and Delta *K*, were observed when *K* was assessed as 3 (Mean LnP [*K*]: –3172.6; Delta *K*: 36.77); Fig. 3B. The results, as shown in Fig. 3C and described by Evanno et al. (2005) consist of plots illustrating the posterior probabilities for the *K* = 3 genetic cluster. The NJ tree also revealed the presence of 3 clusters. Eleven populations showed nearly full membership in one of the 3 genetic clusters, whereas the remaining 3 populations showed a mixed membership with 3 genetic clusters (Table 6).

## Discussion

The analysis of the population genetics of tropical bed bug *C. hemipterus* in Iraq based on genotyping 140 individuals from 14 populations using 7 microsatellite loci was successfully applied for analysis of genetic diversity, and the population dynamics of tropical bed bug in Iraq.

The presence of heterozygous alleles in all samples, as evidenced by the peaks observed in the electropherograms, highlights the genetic diversity within the studied populations. The polymorphism exhibited by the 7 microsatellite loci, with alleles ranging from 6 to 18 per locus (Table 3), underscores the variability present across populations. This finding is consistent with previous population genetic investigations conducted on the tropical bed bug *C. hemipterus* in Malaysia (Seri Masran and Ab Majid 2019, Wan Mohammad et al. 2020).

The mean observed heterozygosity (*H*_obs_) of 0.730 suggests a relatively high level of genetic variation within individuals. In contrast, the expected heterozygosity (*H*_exp_) 0.175 reflects the genetic diversity anticipated under Hardy–Weinberg equilibrium (Table 4). This disparity suggests that the actual genetic diversity within the populations is much higher than what would be expected under Hardy–Weinberg equilibrium. This difference can arise due to several factors that influence population dynamics, like natural selection, genetic drift in smaller populations, or migration of individuals between populations (Gaggiotti et al. 2009).

The mean PIC value of 0.705 (Table 5) indicates that the microsatellite markers used in the study are highly informative and capable of discriminating between different alleles. The average *F*_IS_ of 0.755 (Table 5) suggests a moderate level of inbreeding within populations. The combination of high PIC values and moderate *F*_IS_ values suggests that while genetic diversity exists, there is also evidence of some level of inbreeding. This is further supported by the *F*_IS_ value of 0.761, highlighting the potential mating patterns, and genetic relatedness within populations. The low mean value of genetic differentiation (*F*_ST_ = 0.045) among populations and the low relatedness coefficient of *r* (0.051) emphasize that gene flow between populations is significant despite the potential for inbreeding. The heightened likelihood of inbreeding can be attributed to several factors, such as pest control, human-mediated movement, and infrequent additional introduction events per infestation (Booth et al. 2015).

The AMOVA result (Fig. 2) indicated that 93% of the genetic variation exists within populations, while only 7% is attributed to differences between populations. This finding suggests genetic diversity exists within population rather than between different populations. The NJ tree analysis (Fig. 3A) further supports this, with the populations forming 3 clusters with high bootstrap values. The previous study, done by Seri Masran and Ab Majid (2019) regarding the population genetic structure and breeding pattern in Malaysia supports our results. The formation of 3 distinct genetic clusters suggests regional genetic structuring among the *C. hemipterus* populations in Iraq. The AMOVA result and NJ tree analysis combination suggest that geographic or environmental factors might contribute to the observed genetic structuring. The formation of distinct genetic clusters attribute to specific local conditions.

Using the STRUCTURE software to analyze population structure revealed 3 distinct genetic clusters, as supported by high values of posterior probabilities and Delta K (Fig. 3B and C). Similarly, Narain et al. (2015) study on the bed bug *C. lectularius* population in the USA reported 3 genetic clusters. This implies the presence of different genetic groups within the sampled populations. In essence, the STRUCTURE analysis reinforces the patterns observed in the AMOVA and NJ analysis. It provides visualization and quantification of the genetic clusters, offering additional support to the idea of the tropical bed bug *C. hemipterus* population in Iraq exhibiting regional genetic structuring.

Our finding uncovered a mod shift in the 12 out of 14 studied populations of tropical bed bug, indicating a recent bottleneck event. This observation raises the possibility that tropical bed bug populations may have undergone a reduction in effective population size due to eradication efforts, including insecticides or adaption to repeated cycles of inbreeding. Saenz et al. (2012) suggested that it might be possible that Bed bug populations experienced genetic bottleneck events during the control measures such as the use of insecticides.

In conclusion, the tropical bed bug infestation in Iraq can be attributed to low genetic diversity within and between populations. It can be attributed to the mating between more related individuals. In addition, a low genetic diversity suggested 3 genetic clusters found in populations. The passive dispersal and bottleneck events indicate the low level of genetic differentiation observed between populations. Reusing infested furniture and beds, as well as an increase in the number of local travelers, are all examples of human-mediated transport that necessitate consideration in developing management strategies. Further sampling need to be collected from various regions in Iraq for a better understanding of tropical bed bug genetic structure and breeding pattern.

## Data Availability

The datasets generated and/or analyzed during the current study are available in the NCBI repository, with accession numbers from ON989830 to ON989847 and OP035848 to OP035865.
